# Research on Classification of Grassland Degeneration Indicator Objects Based on UAV Hyperspectral Remote Sensing and 3D_RNet-O Model

**DOI:** 10.3390/s24041114

**Published:** 2024-02-08

**Authors:** Eerdoumutu Jin, Jianmin Du, Yuge Bi, Shengli Wang, Xinchao Gao

**Affiliations:** Mechanical and Electrical Engineering College, Inner Mongolia Agricultural University, Hohhot 010018, China; erdmt@emails.imau.edu.cn (E.J.); biyuge163@imau.edu.cn (Y.B.);

**Keywords:** desert grassland, Unmanned Aerial Vehicle, low-altitude remote sensing, deep learning, convolutional neural network

## Abstract

Real-time and high-precision land cover classification is the foundation for efficient and quantitative research on grassland degradation using remote sensing techniques. In view of the shortcomings of manual surveying and satellite remote sensing, this study focuses on the identification and classification of grass species indicating grassland degradation. We constructed a UAV-based hyperspectral remote sensing system and collected field data in grassland areas. By applying artificial intelligence technology, we developed a 3D_RNet-O model based on convolutional neural networks, effectively addressing technical challenges in hyperspectral remote sensing identification and classification of grassland degradation indicators, such as low reflectance of vegetation, flat spectral curves, and sparse distribution. The results showed that the model achieved a classification accuracy of 99.05% by optimizing hyperparameter combinations based on improving residual block structures. The establishment of the UAV-based hyperspectral remote sensing system and the proposed 3D_RNet-O classification model provide possibilities for further research on low-altitude hyperspectral remote sensing in grassland ecology.

## 1. Introduction

Inner Mongolia is an important ecological security barrier in northern China. It is the main grain production base and animal husbandry base in China. Its environment directly affects the development of agriculture and animal husbandry and the ecological security in China [1,2,3]. Due to the arid and semiarid climate conditions, low precipitation, poor soil conditions, and intensive land use practices, the vegetation communities in the Inner Mongolia Yin Mountain desert grassland often undergo changes, resulting in exceptionally complex regional landscape structures [4,5]. Compared to those in typical grassland areas, desert grassland resources are more vulnerable to climate change and human disturbance, and pasture degradation and desertification problems are becoming increasingly serious [6]. Grassland degradation is first manifested as a reduction in the fractional vegetation cover and number of plant species and expansion of the bare soil area, which directly affects the comprehensive utilization level of grassland resources [7,8]. It is very important to study and elucidate the growth status and change trend of vegetation communities in desert grassland for systematic analysis of grassland resource economic characteristics and rational development and utilization.

The desert grasslands in northern China are mainly dominated by small and medium-sized family ranches. Long-term sedentary grazing, extensive management, and regional overgrazing have caused serious damage to the grassland ecosystem [9]. In grazing grasslands, livestock tend to preferentially consume grassland resources with higher palatability, resulting in a loss of biodiversity and a decline in grassland productivity [10,11]. Degraded grassland has sparse vegetation, low grass layer, wide distribution, and various application modes. The traditional manual survey method is time-consuming and laborious and cannot meet the dynamic monitoring needs of large-scale grassland degradation. Although satellite remote sensing has broken through the limitations of manual investigation at the spatial scale, it can only realize the statistical study of large-scale vegetation coverage due to the limitation of spatial–spectral resolution, and there are obvious deficiencies in characterizing vegetation subtypes or small-scale vegetation community structure [12]. The Unmanned Aerial Vehicle (UAV) hyperspectral remote sensing platform has nanoscale spectral resolution and centimeter-level spatial resolution, and has many advantages such as flexible operation, easy data acquisition, and high efficiency [13], which has been gradually applied in the field of grassland ecological information survey [14].

The traditional analysis method has low recognition accuracy for high-dimensional spectral data, and the analysis process is cumbersome, which makes it difficult to meet the actual needs. In recent years, scholars have developed and optimized the convolutional neural network model for the data characteristics and ground object characteristics of remote sensing data sets [15,16]. However, these deep network models have limitations in processing hyperspectral data of degraded grassland vegetation and cannot show the same performance. In addition, the cost of model training is high, which mainly consists of computational resources and time consumption [17,18]. This study takes the desert grassland degeneration indicator objects in Siziwang Banner, Inner Mongolia as the research object. The remote sensing images of desert grassland were collected by a UAV hyperspectral remote sensing system, and the grassland degradation indicator objects were identified and classified by deep learning method. A classification model suitable for the characteristics of UAV hyperspectral remote sensing data set and grassland degeneration indicator objects is established. The high-precision identification and classification of grassland degeneration indicator objects, including group species, indicator species of degradation, wilted grass, and bare soil, were achieved through the utilization of deep learning methods. It is a meaningful exploration of the classification of desert grassland surface based on remote sensing information. This lays the foundation for real-time, efficient, and high-precision ecological information monitoring and statistics for desert grassland, as well as provides reference for the precise implementation of desert grassland ecological restoration programs.

## 2. Materials and Methods

### 2.1. UAV Low Altitude Remote Sensing System

**Figure 1:**
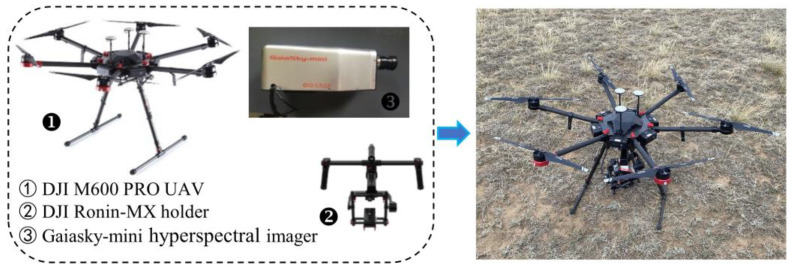
UAV low altitude remote sensing imaging system.

The low-altitude remote sensing system integrated in this research institute mainly consists of a hexacopter drone, a hyperspectral imager, a gimbal, an onboard computer, and other instruments (Figure 1). The six arms of the drone adopt a plug-in structure, which is portable and easy to install. It is equipped with a professional-grade A3 Pro flight control system (Manufactured by DJI Innovation Technology Co., Ltd., Shenzhen, China), three sets of IMU and GNSS modules. The empty weight of the aircraft is 9.5 kg (including the battery), with a maximum payload capacity of 6 kg. The full load flight endurance is 30 min. The information acquisition sensor is the GaiaSky-mini hyperspectral imager (Produced by Sichuan Shuanglihepu Technology Co., Ltd., Chengdu, China), with a spectral range between 400–1000 nm. The size of the spectral image is 696 lines × 775 samples × 256 bands.

### 2.2. Overview of the Study Area

**Figure 2:**
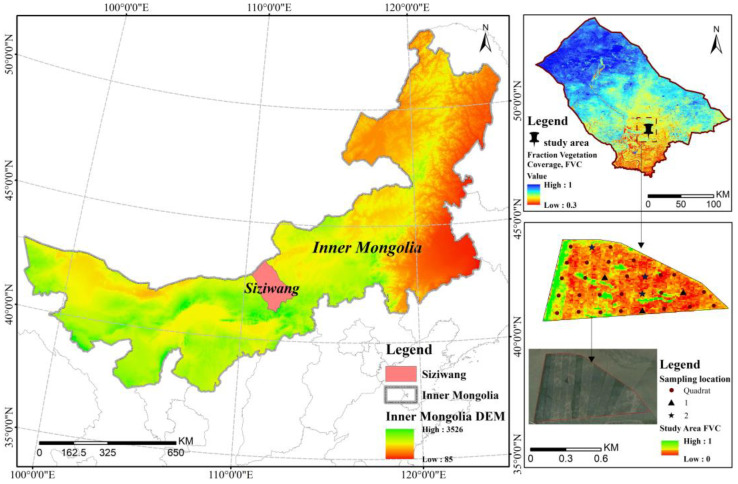
Satellite image of the study area.

The study area is the desert grassland of Siziwang Banner, Yinshan North Foot in Inner Mongolia, with specific coordinates of 41°47′17″ N, 111°53′46″ E and an elevation of 1450 m (Figure 2). It is located in the temperate continental monsoon climate zone, influenced by the Mongolian high-pressure system. The spring and autumn seasons are windy, the summer is dry with little rainfall and large diurnal temperature variations, and the winter is cold. There is a significant amount of wind and sand throughout the year, with more than 50% of days experiencing strong winds. The annual average precipitation is 280 mm, with 80% or more occurring from June to September. Most years experience severe drought. The grassland in this area is a typical desert grassland, characterized by sparse vegetation and low grass height, averaging 8 cm.

The group species in the research area is *Stipa breviflora* Griseb (hereinafter referred to as *S. breviflora*). Group species are the dominant species in the dominant layer of the community, which play a leading role in the community structure and community environment and are the creators and builders of the community. *S. breviflora* possesses strong adaptability to wind and sand, as well as cold, drought, and grazing resistance. The succession of vegetation community structure is a direct manifestation of grassland degradation. In recent years, under the dual impact of arid climatic conditions and excessive grazing, the original vegetation community structure in the research area has been destroyed, and the *Artemisia frigida* Willd community gradually becomes the dominant species in the vegetation community structure (hereinafter referred to as *A. frigida*). Therefore, the *A. frigida* community is recognized as an indicator species of grassland degradation [19,20]. Indicator species for degradation can be defined as plant species with degradation indication significance, which plays an important role in the process of grassland degradation succession. Therefore, the accurate identification of plants such as *S. breviflora* and *A. frigida* is extremely important in the study of indicators of desert grassland degradation. In this study, *S. breviflora*, *A. frigida*, withered grass, and bare soil were selected as the research objects for grassland degeneration indicators. After field visits and geographical information gathering, a natural grassland area of 13.2 hm^2^ was delineated as the unmanned aerial vehicle flight area.

### 2.3. Data Collection

**Figure 3:**
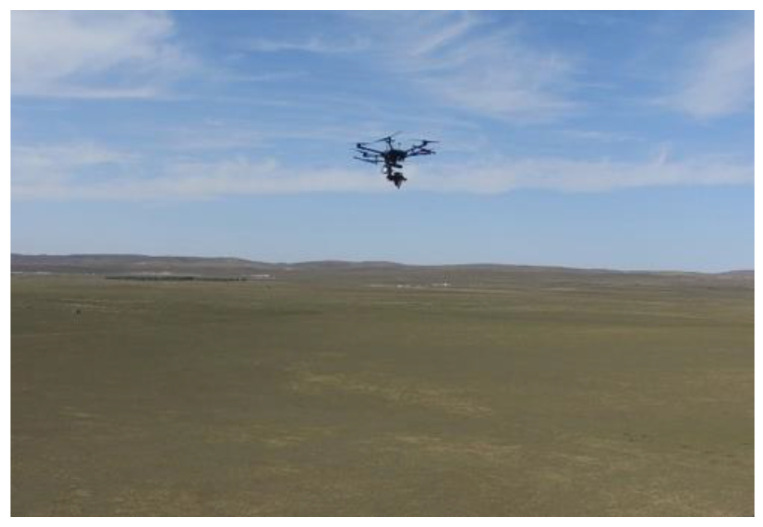
UAV hyperspectral system data collection.

According to the climate characteristics of degradation indicator species in 2022 and the growth cycle characteristics of grass, the experimental group conducted detailed investigations in the experimental area during the period of June to August 2022, when the plant growth was most vigorous. A random block design was employed to establish a total of 30 pure plots in the relatively aggregated growth areas of *S. breviflora* and *A. frigida* communities (Figure 2), with each type comprising 15 plots. Considering the topography and vegetation growth characteristics of the study area, the types of *S. breviflora* and *A. frigida* quadrats were determined by the types of grasses with more than 98% of the total grass in the quadrats. The left and right spacing of the random quadrats was about 15 m, and the front and back spacing was about 10 m. The size of the hyperspectral image of the collected quadrats was 696 line × 775 sample × 256 band. To ensure the imaging quality of the low-altitude UAV hyperspectral remote sensing images, data collection was carried out under meteorological conditions with wind speeds lower than Grade III (≤5.4 m/s) and clear skies or cloud cover below 2% during the period of maximum solar altitude from 10:00 to 14:00. The UAV hyperspectral imaging system can achieve route flight, with flight routes planned from the ground station (Figure 3). Each plot was photographed no less than 5 times to improve the availability of the data. The UAV flew at a height of 20 m and a speed of 1 m/s, with a lateral overlap set at 55%. The image spectral resolution was 2.6 nm, and the spatial resolution was 1.73 cm/pixel.

### 2.4. Creating a Dataset

**Figure 4:**
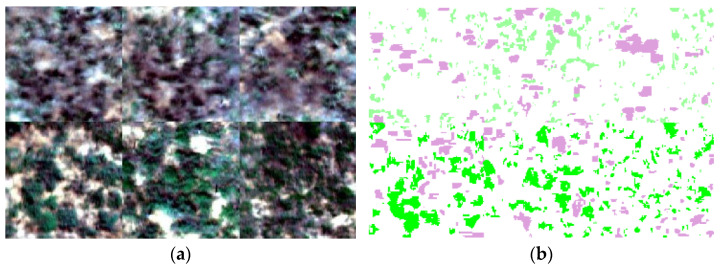
Indicator species for degradation dataset. (**a**) False-color composite image. (**b**) Label true value.

**Figure 5:**
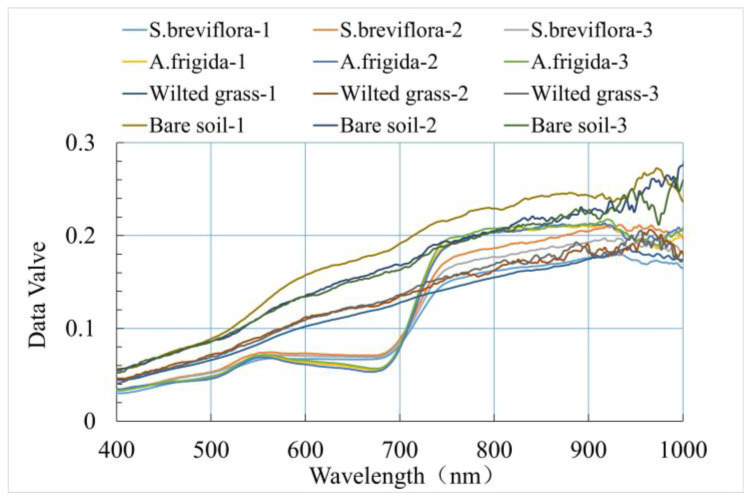
Spectral reflectance of grassland degradation indicator objects.

Human inspection was carried out to address the poor imaging quality of remote sensing images caused by variations in light intensity and gusty winds. Subsequently, radiometric correction was performed using Spec VIEW2.9 software. The single image after radiation correction occupies 1.2 GB of storage space. In order to establish a more comprehensive and abundant spectral information database and improve the computational efficiency of the network model, in this study, three quadrats were randomly selected from the two categories of *S. breviflora* and *A. frigida* in the hyperspectral remote sensing data of pure quadrats, and the center of each selected quadrat was cropped to 150 line × 150 sample × 256 band. A new data set (450 line × 300 sample × 256 band) was generated by splicing two types of data sets (Figure 4a). A total of 135,000 pixels were used to make the sample hyperspectral database, which can effectively increase the richness and representativeness of the data set and help to improve the robustness of the model. A total of 300 pure pixels were selected from each category’s region of interest and averaged to obtain the reflectance values of different land cover types (Figure 5). The land cover types were classified into three categories: *S. breviflora*, *A. frigida*, and non-vegetation (withered grass, bare soil) markers. In total, 33,582 pixels were labeled as samples (Figure 4b), with a training-to-testing sample allocation ratio of 7:3 (Table 1).

### 2.5. Data Dimensionality Reduction and Patch Segmentation

To eliminate redundant data, this study adopts the most frequently used Principal Component Analysis (PCA) technique to reduce data dimensionality and enhance computational efficiency. After performing PCA computation while retaining 98% of the original data’s information, a total of 30 spectral bands are obtained. Each central pixel and its spatial neighborhood are segmented into 3D data blocks with dimensions of S × S × B, where S × S represents the width and height of the patch size, and B corresponds to the number of spectral bands.

### 2.6. Construction of the DGRNet Model

**Figure 6:**
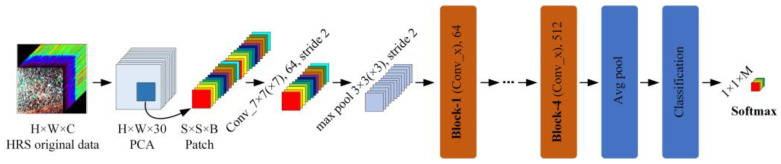
DGRNet model structure diagram.

Grassland degeneration indicates that high-resolution hyperspectral data possess fine-grained and mixed-pixel characteristics, making information extraction and resolution exceptionally challenging. To address this issue, traditional machine learning methods, such as Support Vector Machines (SVM), construct hyperplanes by projecting features into high-dimensional space to accomplish classification objectives. However, this approach heavily relies on manual feature extraction, requiring extensive expert knowledge and parameter settings. In contrast to machine learning feature engineering methods [21,22], deep learning has the advantage of extracting intrinsic deep features from images, which is beneficial for resolving the classification problem of desert grassland degradation indicators. In order to better evaluate the performance of 2D convolution and 3D convolution in convolutional neural networks, this study builds upon a constructed simplified 2D ResNet model (DGRNet) and further establishes a 3D_ResNet model (3D_DGRNet). The specific diagram illustrating the model structure can be found in Figure 6.

In the figure, H, W, and C represent the row height, column width, and band number of the data set, respectively, S represents the patch size, and B represents the patch size band number. After the first layer convolution operation of the model, max pool processing is performed. The size of conv (1) is set to 7 × 7 (×7), the size of max pool is set to 3 × 3 (×3), and the stride is set to 2, respectively. Block 1–4 represents the residual block of the deep neural network, respectively, and Batch Normalization and ReLU activation functions are used after convolution operation in each layer of the residual block. The convolution kernel size of the model is 3 × 3 (×3), and the spatial size of the input is 9 × 9. The batch sizes are 64, 128, 256, and 512, respectively. After the convolution, the global average pooling function is used to convert the output feature map into a fixed-length vector representation. Finally, through the Softmax function, the original output is converted into a probability distribution, that is, the probability of each category.

### 2.7. Constructing the 3D_RNet-O Model

By establishing a deep learning network model, the performance advantages of convolutional neural networks in desert grassland degradation indicator species monitoring were verified, indicating the feasibility of deep learning for identifying indicator species of desert grassland degradation. Therefore, this study will improve the 3D_DGRNet model based on this foundation to further enhance the recognition accuracy of deep learning models for indicator species of desert grassland degradation.

**Figure 7:**
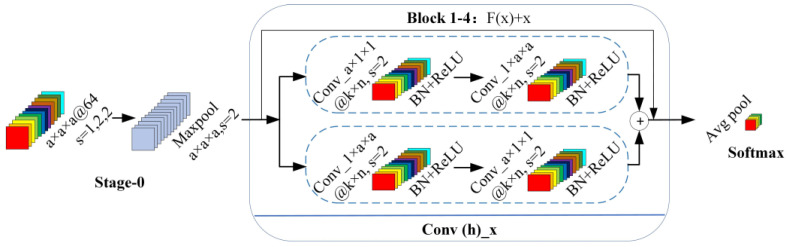
Schematic diagram of 3D_RNet-O model structure.

The 3D-ResNet model is typically composed of multiple stacked residual blocks, each containing several convolutional layers. In existing research models, symmetric convolution and single-scale convolution are commonly used as residual blocks, which have limited ability to handle the variations in different scales and local details of multi-channel processing objects. Moreover, using the same size of convolution kernels restricts the receptive field range, leading to the loss of important detailed information during the convolution process. Therefore, this study proposes an improved 3D_DGRNet model called 3D_RNet-O (Figure 7).

The design of the 3D_RNet-O model takes into consideration the scale variations and local details of multi-channel processing objects. The residual block structure mainly includes designs such as dual-branch features and multi-scale convolution fusion. In this study, each convolution layer of the residual block is decomposed into a spectral convolution block and a spatial convolution block. The spectral–spatial convolution blocks are then concatenated, followed by feature fusion of the two branches. This allows for the concatenation of multi-scale local features, preserving more detailed information. In the 3D_RNet-O model, a composite function is used with two different sizes of 3D convolution kernels: a × 1 × 1 and 1 × a × a. The first branch employs spectral convolution blocks (a × 1 × 1) and spatial convolution blocks (1 × a × a), while the second branch uses spatial convolution blocks (1 × a × a) and spectral convolution blocks (a × 1 × 1). This establishes a dual-branch feature learning model and concatenates the learned features from both branches.

### 2.8. Accuracy Evaluation of the Model

To evaluate the classification accuracy of the monitoring model, this study will use overall accuracy (OA), average accuracy (AA), and Kappa coefficient as evaluation metrics to analyze the model’s performance. OA refers to the proportion of correctly classified pixels in the total number of pixels in random samples, which provides a more intuitive reflection of the accuracy of hyperspectral remote sensing classification results. AA refers to calculating the classification accuracy of each category and averaging the accuracy of all categories in a multi-classification task, which can provide a more comprehensive assessment. The Kappa coefficient is an evaluation index based on the confusion matrix that measures the reliability of the classification results. It effectively addresses the issue of small sample sizes and provides a more comprehensive reflection of the classification performance.

## 3. Results

### 3.1. Platform and Network Training

The training process in this study was conducted using the following platform: i7-6820 HK CPU, with a memory capacity of 32 GB, and Nvidia Geforce GTX 1080, an 8 GB GPU. The training iteration was set to 200. All the accuracy values in this section are the average values obtained from 10 repeated experiments of the model.

To objectively evaluate the effectiveness of the proposed methods in this study, four models were trained: SVM, DGRNet, 3D_DGRNet, and 3D_RNet-O. The SVM model uses the Radial Basis Function (RBF) as its kernel function. DGRNet and 3D_DGRNet are classic ResNet models. 3D_RNet-O is a ResNet model based on the 3D_ResNet backbone network. In the 3D_RNet-O model, the convolution layers in the residual block are decomposed into spectral–spatial convolution blocks, and detailed features are extracted using dual-branch operations.

### 3.2. Classification Results of the Baseline Models

**Figure 8:**
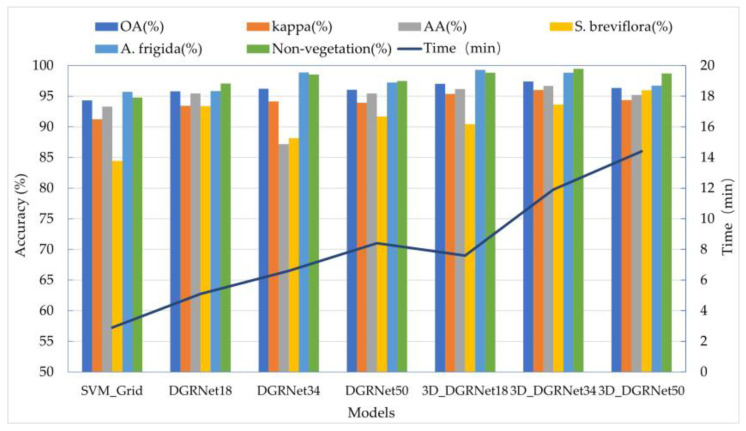
Comparison of recognition accuracy of different network models.

Trained by the initial classification models, the 3D_DGRNet model exhibited superior classification performance, followed by the DGRNet model, while SVM_Grid performed the worst (Figure 8). The 3D_DGRNet model incorporates both 3D convolution and 2D convolution operations to consider the spectral–spatial feature relationship in the dataset, enabling effective utilization of spatial and spectral information. Compared to 2D convolution kernels, 3D convolution kernels significantly improved the recognition accuracy of indicator species of degradation. By increasing the network depth, the ResNet model enhances the representational and learning capabilities of the model. Therefore, in this study, different network depths were selected for the DGRNet and 3D_DGRNet models, including 18 layers, 34 layers, and 50 layers. The results demonstrate that when the network depths were, respectively, set to 18, 34, and 50, with a Patch size of 9, a learning rate of 0.003, and batch sizes of 64, 128, 256, and 512, the obtained overall accuracies were 95.78%, 96.21%, 96.07%, 97.02%, 97.41%, and 96.33%, respectively.

In the task of monitoring indicator features of degradation in grasslands, analysis reveals that the 34-layer model achieves the highest classification accuracy of 97.41%. The 18-layer model also performs well with an accuracy of 97.02%. Although the 50-layer model shows a slight decrease in performance, it still maintains good accuracy at 95.33%. Considering both performance and complexity, selecting the 34-layer 3D_DGRNet model as the preferred model is a reasonable choice as it strikes a good balance between performance and complexity. Therefore, based on this, an improved high-accuracy 3D_RNet-O model is proposed for the monitoring of indicator features of degradation in desert grasslands. By adjusting various hyperparameters such as learning rate, batch size, max pooling, and initial convolution operations according to parameter logic relationships and experimental design principles, the 3D_RNet-O model is further enhanced.

### 3.3. Classification Results of 3D_RNet-O Model

To evaluate the performance of the 3D_RNet-O model more effectively, we trained the model while keeping the base network’s hyperparameters such as patch size, learning rate, and batch size constant. At the same time, user accuracy (UA) and producer accuracy (PA) are selected as the accuracy evaluation indexes of different categories. The confusion matrix of classification results is shown in Table 2. UA refers to the ratio of the number of samples correctly classified in each category to the total number of samples in that category. PA refers to the ratio of the number of correctly classified samples to the total number of samples actually belonging to the category. From the perspective of PA, except for *S. breviflora*, the recognition accuracy of other categories reached more than 98%, indicating that the model had less misclassification of *A. frigida* and non-vegetation communities. From the perspective of UA, a small part of *S. breviflora* was identified as non-vegetation community, resulting in a low UA value of non-vegetation community, which was mainly due to the presence of a large number of withered grass at the junction of *S. breviflora* and non-vegetation community. From the overall performance of the model, OA reached 98.21%, and Kappa coefficient reached 0.979.

#### 3.3.1. Learning Rate Optimization

**Figure 9:**
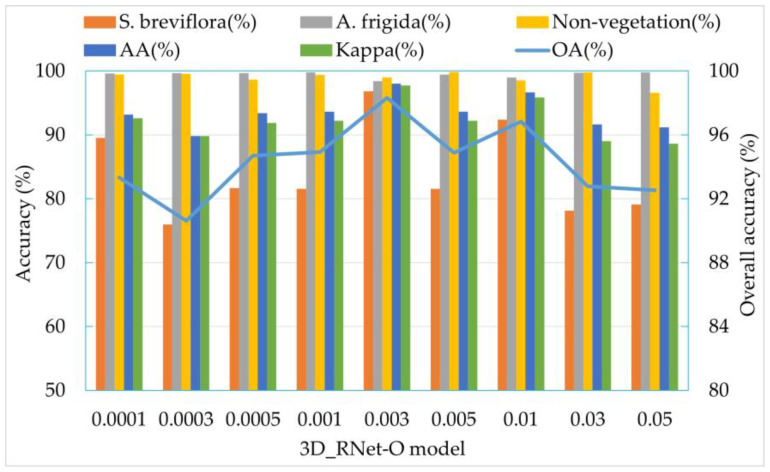
Optimization results of the learning rate.

On the basis of keeping all other hyperparameters unchanged in the 3D_RNet-O model, the learning rate was varied for performance evaluation. Nine different learning rates were tested: 0.0001, 0.0003, 0.0005, 0.001, 0.003, 0.005, 0.01, 0.03, and 0.05 (Figure 9). When the learning rate is set to 0.003, the model achieves optimal recognition performance, with Kappa coefficient and overall accuracy (OA) reaching their highest values at 97.72% and 98.32%, respectively. The accuracy of the *S. breviflora* community, which initially had a slightly lower precision, increased by 2.34%. However, when the learning rate is adjusted to 0.05, despite achieving the best classification performance for the *A. frigida* community, with a classification accuracy of 99.81%, the classification accuracy for the *S. breviflora* community drops to 79.11%. It is evident from the comparison that when the learning rate exceeds 0.003, the model’s performance deteriorates, as a higher learning rate prevents the model from attaining the global optimum.

#### 3.3.2. Optimization of Batch Size

Through batch size optimization, it is possible to standardize the input data for each batch, which helps improve network stability and accelerate convergence speed. The learning rate was set to 0.003, and on the basis of keeping all other hyperparameters of the network model unchanged, the batch size for each residual block was optimized in three sets of experiments, labeled as groups a, b, and c. (Table 3). The experiments show that with the increase in batch size, the model’s classification performance improves significantly. In experiment c, when the batch sizes are set to 128, 256, 512, and 1024, all metrics show improvement, leading to optimal classification performance of the model. Therefore, the batch size options can be 128, 256, 512, and 1024. The testing accuracy of *S. breviflora* has increased to 97.75%, while the accuracies of the other two classes remain relatively stable, with *A. frigida* and non-vegetation achieving testing accuracies of 99.48% and 97.95%, respectively. The overall accuracy has increased by 0.21%.

#### 3.3.3. Optimization of Training Sample Proportion

**Figure 10:**
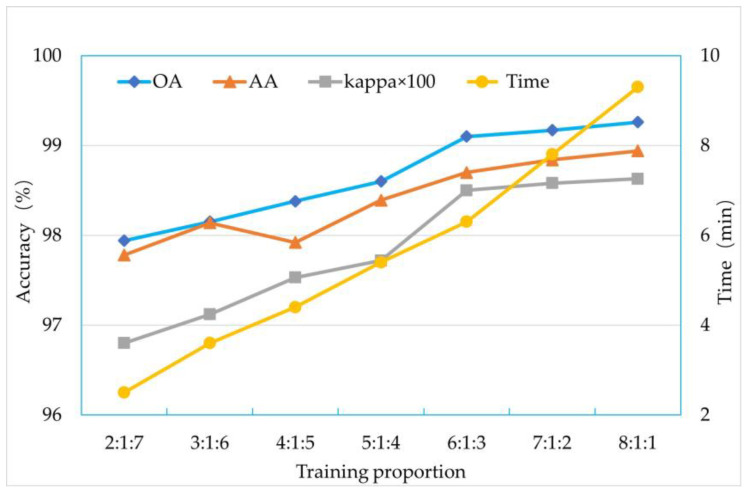
Proportional optimization calculation results of training samples.

In model training, the setting of training sample ratios has a significant impact on prediction results. When the training sample ratio is low, the model can only learn the representation of fewer land covers, thus failing to predict land covers with greater variations effectively. Conversely, when the training sample ratio is high, the model will have more opportunities to learn the representation of various land covers and make more accurate predictions. However, setting the training sample ratio too high will lead to a sharp increase in training time, with potentially marginal improvements in prediction accuracy. In this experiment, the training sample ratio was set to increase by 10%, starting from 20% and gradually increasing to 80%, with a total of seven experiments. In each experiment, the validation ratio was consistently set at 10% (Figure 10). It can be observed from the graph that as the training data increase, the required training time also increases correspondingly. Under the same number of iterations, the model’s overall accuracy (OA), average accuracy (AA), and Kappa coefficient show an increasing trend with the increase in training samples. However, after reaching a training ratio of 60% (6:1:3), the performance improvement of the model becomes sluggish while the time consumption rises sharply. Therefore, considering all factors, the training sample ratio can be chosen as 60%.

#### 3.3.4. Optimization of other Hyperparameters

**Figure 11:**
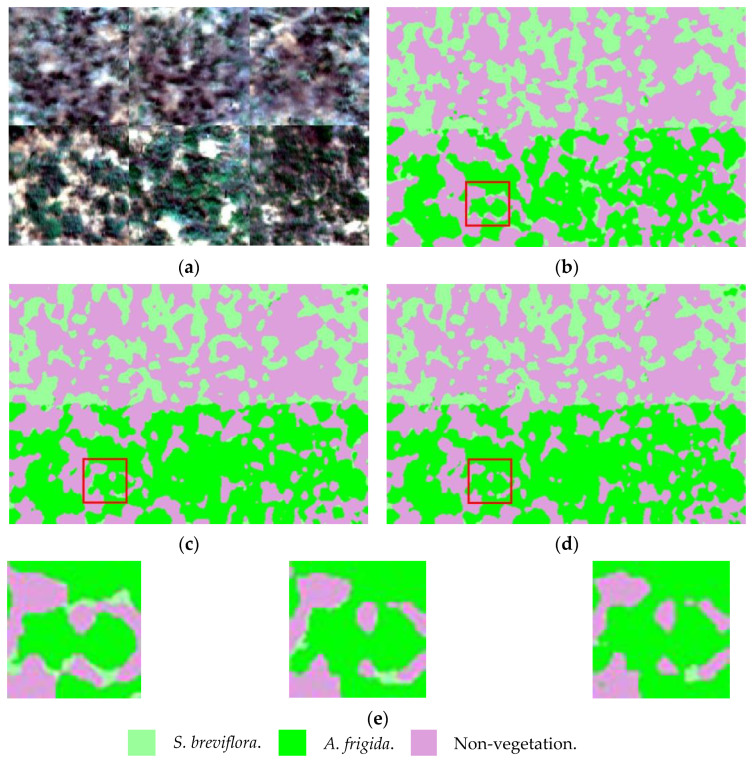
Visualize the classification results of different models. (**a**) False-color composite image. (**b**) DGRNet34. (**c**) 3D_DGRNet34. (**d**) 3D_RNet-O. (**e**) Enlargement of the red boxed area in figures (**b**–**d**).

After changing the pooling window of the 3D_RNet-O model’s max pooling operation from (3,3,3) to (1,1,1) and (2,2,2) for training, the results showed that the (2,2,2) window had a more noticeable advantage in group species recognition. Changing the initial convolution operation Conv_7,7,7 to (5,5,5), (3,3,3), (3,5,5), and (5,7,7) for training showed that when using Conv_3,5,5, there was a slight improvement in the model’s performance. Under the optimal hyperparameter combination, the confusion matrix of the 3D_RNet-O model is presented in Table 4. From the perspective of PA, the accuracy of different categories all exceeds 98%. Compared with the parameter optimization before, the model’s feature extraction capability for each category has significantly improved. From the perspective of UA, the testing accuracy for *S. breviflora* is 97.34%, while the testing accuracies for the other categories exceed 99%, indicating that the network possesses effective feature extraction capabilities for grassland degeneration indicators. The comparison of visualization results between the base model and 3D_RNet-O model can be seen in Figure 11.

**Table 4:** Classification confusion matrix of optimal parameter combination.

Category	*S. breviflora*	*A. frigida*	Non-Vegetation	PA (%)
*S. breviflora*	1900	6	14	98.96
*A. frigida*	36	3696	12	98.72
non-vegetation	16	0	3390	99.53
UA (%)	97.34	99.84	99.24	

#### 3.3.5. K-Cross Validation (K-CV)

In deep learning, accurately evaluating the performance of a network model on an unseen dataset is crucial. Cross Validation is a statistical analysis method used to validate the performance of classifiers. It involves training the model on the training set and then comparing the model’s predictions with the validation set, ultimately using accuracy as the performance metric for the classifier. K-CV involves dividing the dataset into K groups and then taking each group in turn as the testing set, while using the remaining K-1 groups as the training set. The average accuracy of the K training–testing iterations is then used as the evaluation metric for the classifier’s performance. Compared to other Cross Validation methods, K-CV has the advantage of more effectively preventing overfitting and underfitting, which leads to higher credibility in simulating results. In this section, the values of K are set to 3, 5, 7, and 9, and the classification accuracy and variance corresponding to each K value are obtained through experiments (Table 5). The experimental results indicate that as the value of K increases, the stability of the classification results gradually improves, and when K reaches 7, the variation in the classification results tends to level off. The average variance of the classification accuracy is only 0.009. This finding demonstrates the effectiveness of the 3D_RNet-O model and the high reliability of the results.

#### 3.3.6. Ablation Experiment

To verify the effectiveness of different modules in the 3D_RNet-O model, this section conducted ablation experiments for analysis (Table 6). From the results of experiment a, it can be observed that the model with only single 3D convolution feature extraction has the poorest classification accuracy. When the first branch (a × 1 × 1, 1 × a × a) or the second branch (1 × a × a, a × 1 × 1) is added, the model’s classification performance shows a significant improvement, indicating that the combination of spectral convolution block and spatial convolution block in the residual block can effectively enhance the model’s classification performance. In experiment d, when the dual-branch module is simultaneously added to the residual block, the model’s classification performance is greatly improved. Considering the overall performance indicators of the model, the OA, AA, and Kappa have, respectively, increased by 1%, 1.66%, and 0.019. This further demonstrates that the 3D_RNet-O model can more effectively extract the joint spectral–spatial information from remote sensing images from a global perspective.

### 3.4. Comparison of Computational Efficiency and Model Parameters

To further discuss the parameter size and computational efficiency of the 3D_RNet-O model, we conducted experimental analysis on models with the same network depth (Table 7). SVM has fewer hyperparameters, resulting in the lowest time cost, but its classification performance is relatively poor. Among the convolutional neural network-based classification models, DGRNet has the lowest total parameter count and model size, as well as lower time cost, but its overall classification performance is inferior. In comparison to the 3D_DGRNet model, the proposed 3D_RNet-O model demonstrates a significant reduction in parameter count and model size, making it easier for deployment on mobile devices. This indicates that the 3D_RNet-O model not only ensures classification accuracy, but also exhibits higher computational efficiency and advantages in terms of model size.

## 4. Discussion

The boxed area in Figure 11 shows a magnified view of the visual classification results. A comparison reveals that DGRNet34 fails to accurately identify *A. frigida*, and there is a significant misclassification probability with *S. breviflora*. Although 3D_DGRNet34 successfully identifies some instances of *A. frigida*, it still exhibits misclassifications with *S. breviflora*. Moreover, it struggles to effectively recognize *S. breviflora* and often misclassifies it as non-vegetation. The 3D_RNet-O model performs better than the 3D_DGRNet34 model, as it identifies more instances of *S. breviflora*. It also demonstrates a notable improvement in identifying *A. frigida*, although there are still small areas of misclassification with *S. breviflora*. Through field investigations and Region of Interest (ROI) analysis in ENVI, it was found that the spectral curves of *A. frigida* communities in desert grasslands become flat and featureless due to drought stress. This change makes it difficult to distinguish pixels that are mixed with *S. breviflora* in the image, leading to issues such as same-spectrum foreign bodies and homogeneous spectra [23,24]. This is also a challenging task in the current research on desert grassland land cover classification and inversion and has become a key technical challenge.

The current low-altitude remote sensing data of drones have been widely applied in research areas such as farmland and forests with fixed and regular shapes. However, there is relatively little research on the degraded grassland landscape that consists of small and sparsely distributed land features [25,26,27,28]. In recent years, significant progress has been made in grassland monitoring research based on digital cameras and multispectral data. However, the focus has mainly been on aspects such as fractional vegetation cover, above-ground biomass, vegetation moisture content, average community height, and the impact of different grazing intensities on grassland biomass [14,22,29]. However, research on the identification of degraded grassland vegetation communities is still in its early stages. Existing studies mainly focus on the classification and identification of species such as bare soil, grass, and shrubs [18,21], The UAV hyperspectral remote sensing system and deep learning techniques employed in this study provide high spectral–spatial resolution, enabling effective and accurate extraction of detailed texture features of desert grassland vegetation communities.

Currently, breakthroughs have been made in the exploration of spectral information, spatial information, and spectral–spatial joint information using deep learning methods. Scholars have proposed their own models and solutions. In the research of spatial feature extraction and classification, researchers have used models such as ResNet, VGGNet [30,31], and Densenet [32,33] to significantly improve classification accuracy. However, the accuracy of complex land object recognition is relatively low [34]. Scholars have used 3D-CNN models to classify land objects with large differences in spectral reflectance, such as grass vegetation, bare soil, and markers, achieving higher classification accuracy [24]. By combining 1D-CNN and 2D-CNN, scholars have simultaneously extracted spectral and spatial features, effectively enhancing feature extraction capabilities. However, the process is cumbersome and the improvement in classification accuracy is limited [35]. To address problems such as redundant information in adjacent spectral bands of hyperspectral images, incomplete information feature extraction between spectral dimensions in 2D-CNN, and high computational complexity of 3D-CNN, scholars have proposed a fusion of vegetation indices and 3D-2D-CNN classification method, which effectively enhances feature extraction capabilities. However, there are issues such as high computational pressure and increased training costs [17,36]. More research shows that 3D-CNN has advantages in joint extraction of spectral–spatial features [24,36] and is more suitable for hyperspectral feature extraction. Based on 3D-ResNet, this study proposes the 3D_RNet-O improved model, which has objective training costs and high recognition and classification accuracy for small target objects. It has significant advantages in monitoring applications of indicators of grassland degradation.

## 5. Conclusions

This research focuses on the distribution characteristics of indicator objects for desert grassland degradation and integrates an unmanned aerial vehicle hyperspectral remote sensing system. For the first time, hyperspectral data collection of indicator objects for grassland degeneration in northern desert grasslands is conducted. A vegetation community classification model based on 3D_RNet-O is proposed to classify grass species groups indicating grassland degeneration, degraded indicators, withered grass, bare soil, etc., achieving high accuracy.

In the process of training the basic model, it was found that deep learning has more potential than machine learning in extracting the latent information contained in high-dimensional datasets of hyperspectral remote sensing for degraded grassland vegetation. In order to address the issue of poor identification results caused by flat spectral curves and weak characteristics in degraded grassland, the recognition rate of group species can be effectively improved through improvements in the convolutional layer structure in residual blocks. By continuously adjusting and optimizing hyperparameters such as the size of convolutional kernels, learning rate, batch size, training ratio, and max pooling, the overall classification accuracy can reach up to 99.05%.

## Figures and Tables

**Table 1:** Color coding and sample number.

	Class	Color	Training	Test	Total
1	*S. breviflora*		5056	2168	7224
2	*A. frigida*		9596	4112	13,708
3	Non-vegetation		8856	3794	12,650
	Total		23,508	10,074	33,582

**Table 2:** Confusion matrix based on initial 3D_RNet-O model classification.

Category	*S. breviflora*	*A. frigida*	Non-Vegetation	PA (%)
*S. breviflora*	1815	32	73	94.51
*A. frigida*	16	3723	5	99.44
non-vegetation	39	0	3367	98.86
UA (%)	97.05	99.15	95.6	

**Table 3:** Table of batch size optimization for 3D_RNet-O model.

No.	Block_0	Block_1	Block_2	Block_3	Block_4
a	Conv_@64	Conv_@32	Conv_@64	Conv_@128	Conv_@256
b	Conv_@64	Conv_@64	Conv_@128	Conv_@256	Conv_@512
c	Conv_@64	Conv_@128	Conv_@256	Conv_@512	Conv_@1024

**Table 5:** K-CV experimental results.

K	3	5	7	9
Accuracy (%)	98.85	98.94	98.91	98.96
Accuracy variance (%)	0.059	0.012	0.009	0.008

**Table 6:** Analysis results of ablation experiments.

No.	3D_Conv	First Branch	Second Branch	OA (%)	AA (%)	Kappa
a	√			97.41	96.69	0.958
b	√	√		97.82	96.78	0.959
c	√		√	98.05	97.26	0.962
d	√	√	√	99.05	98.92	0.981

**Table 7:** Computational efficiency and model parameters of different models.

Models	SVM	DGRNet	3D_DGRNet	3D_RNet-O
Total params	/	21,370,883	63,471,171	28,357,699
Parameters size (MB)	/	81.5	242.1	108.2
Time (s)	144.3	396.1	714.4	402.7

## Data Availability

The data presented in this study are available on request from the corresponding author. The data are not publicly available due to data privacy.
